# Supplementary material for: Dynamic Model for RNA-seq Data Analysis
Source: Biomed Res Int. 2015 Aug 4;2015:916352. doi: 10.1155/2015/916352 (PMC4539434; doi:10.1155/2015/916352)
Supplement: Supplementary file 1 — The transient response of the dynamic systems is an important property of the system itself. It can be used to quantify the space domain characteristics of the gene regulation system responding to the disturbance of environments. Our goal is to investigate how the gene expression level at each genomic position varies in response to the external perturbation and whether this will affect the function of cell. Figures S1A-D and Figures S2A-D plotted the average expression curves, unit-step response curves, the coefficient curves of the ODE of genes gene ABHD10 and BTS2, respectively. [file 916352.f1.pdf]

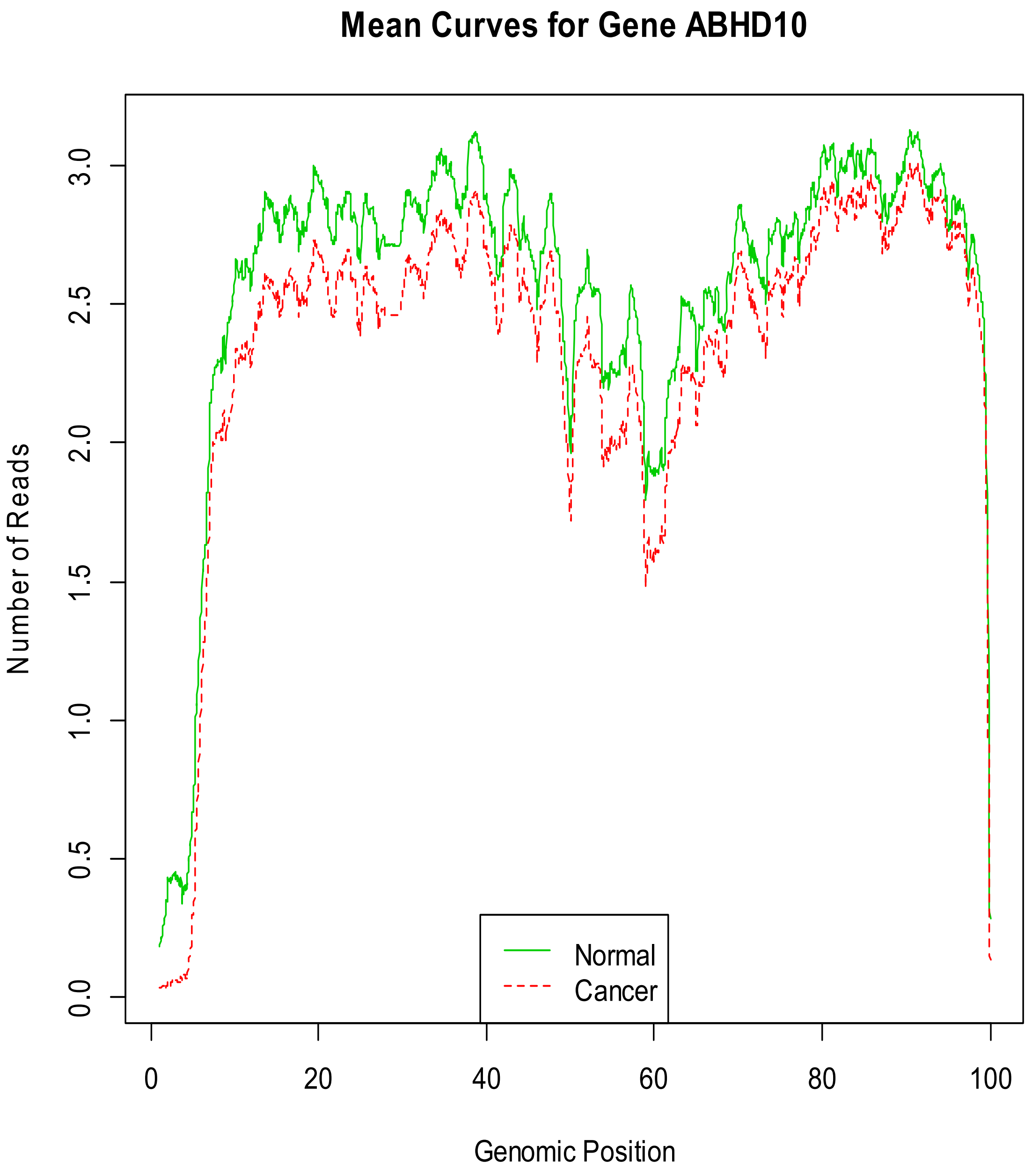

Fig S1A. Average expression curves of gene ABHD10 in the tumor and normal samples.

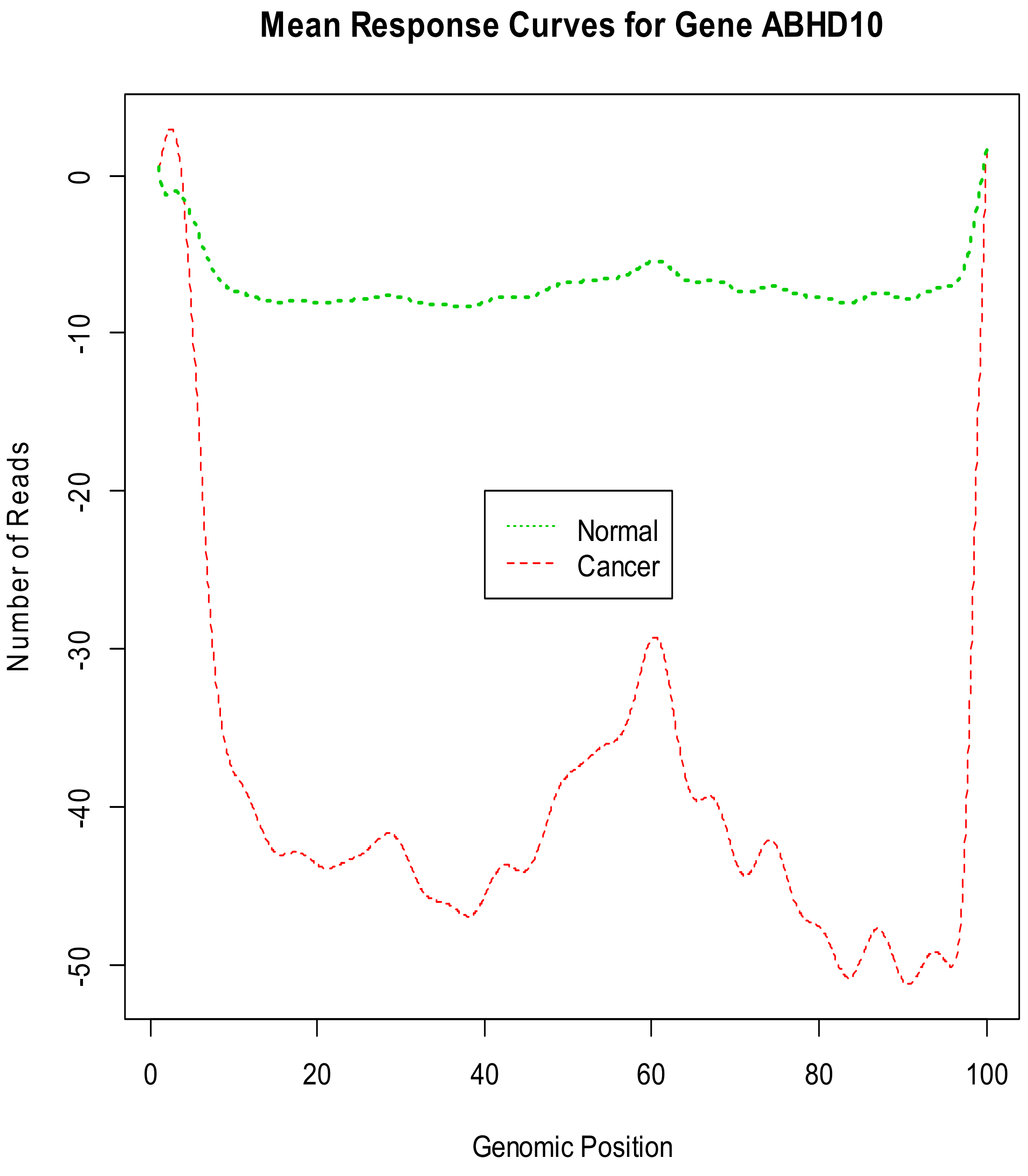

Fig S1B. Average unit-step response curves of gene ABHD10 in the tumor and normal samples.

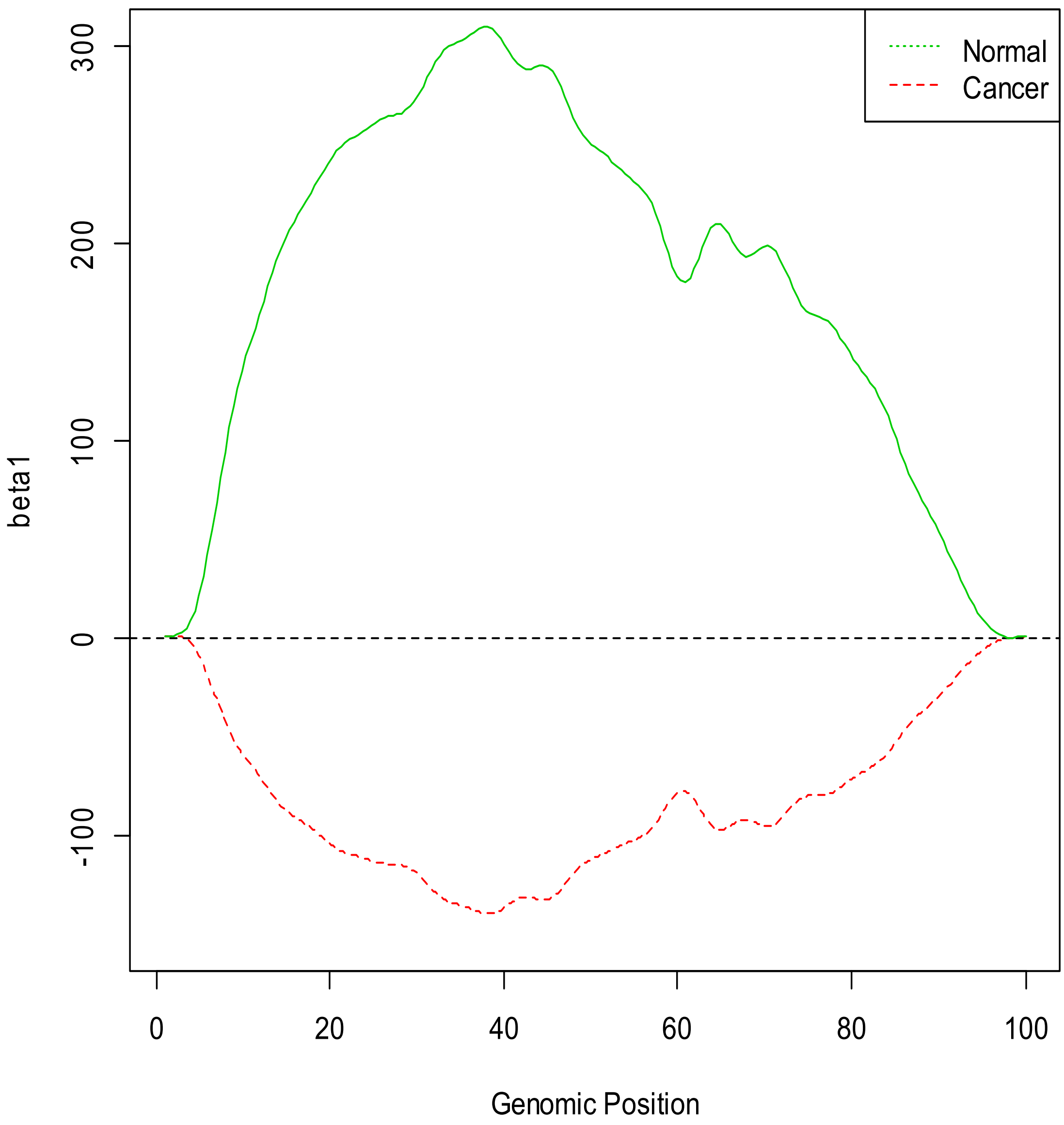

Fig. S1C. Average coefficient curve $\beta_1(t)$ of the ODE for gene ABHD10.

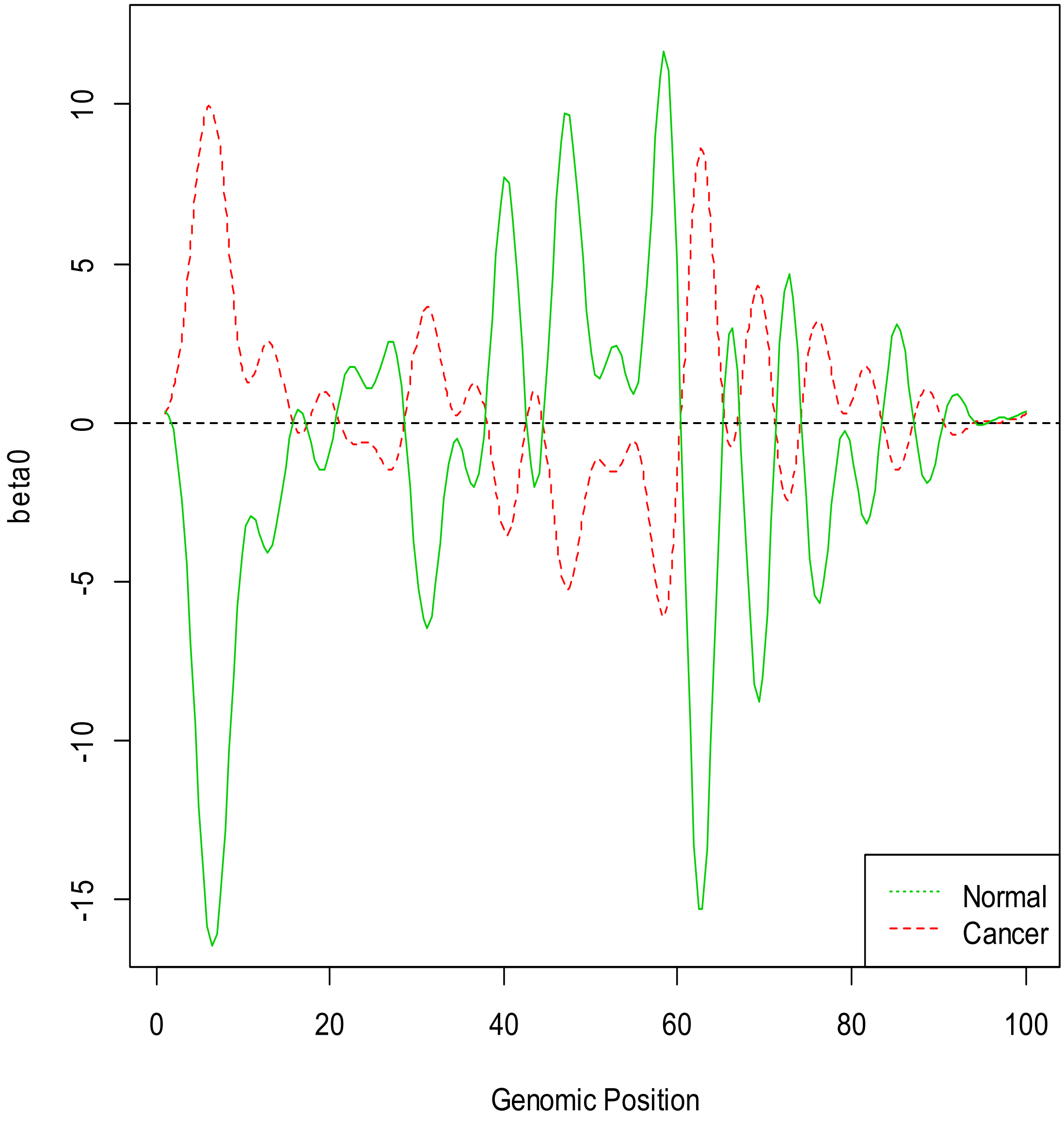

Fig. S1D. Average coefficient curve $\beta_0(t)$ of the ODE for gene ABHD10.

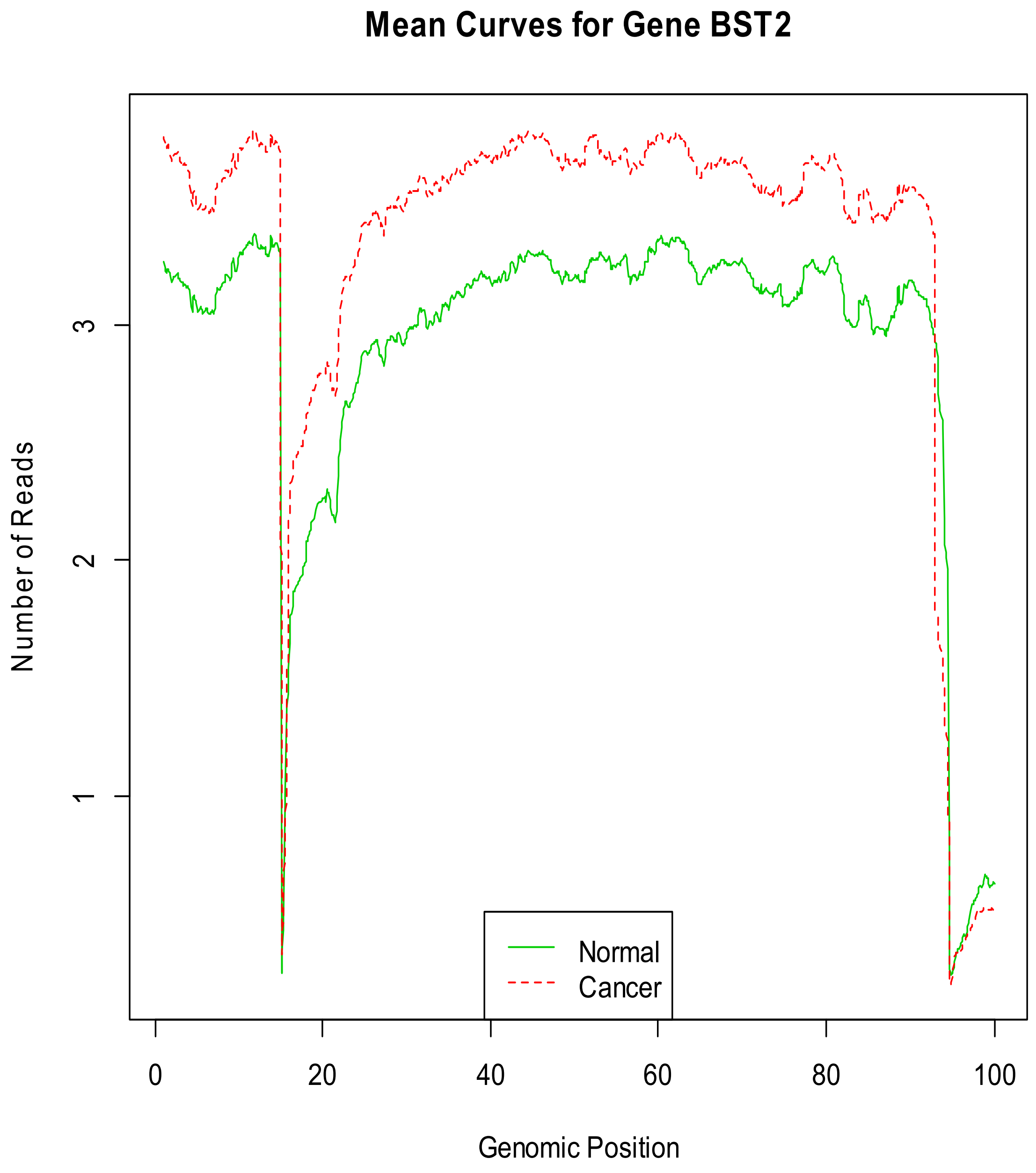

Fig. S2A. Average expression curves of gene BST2 in tumor and normal samples.

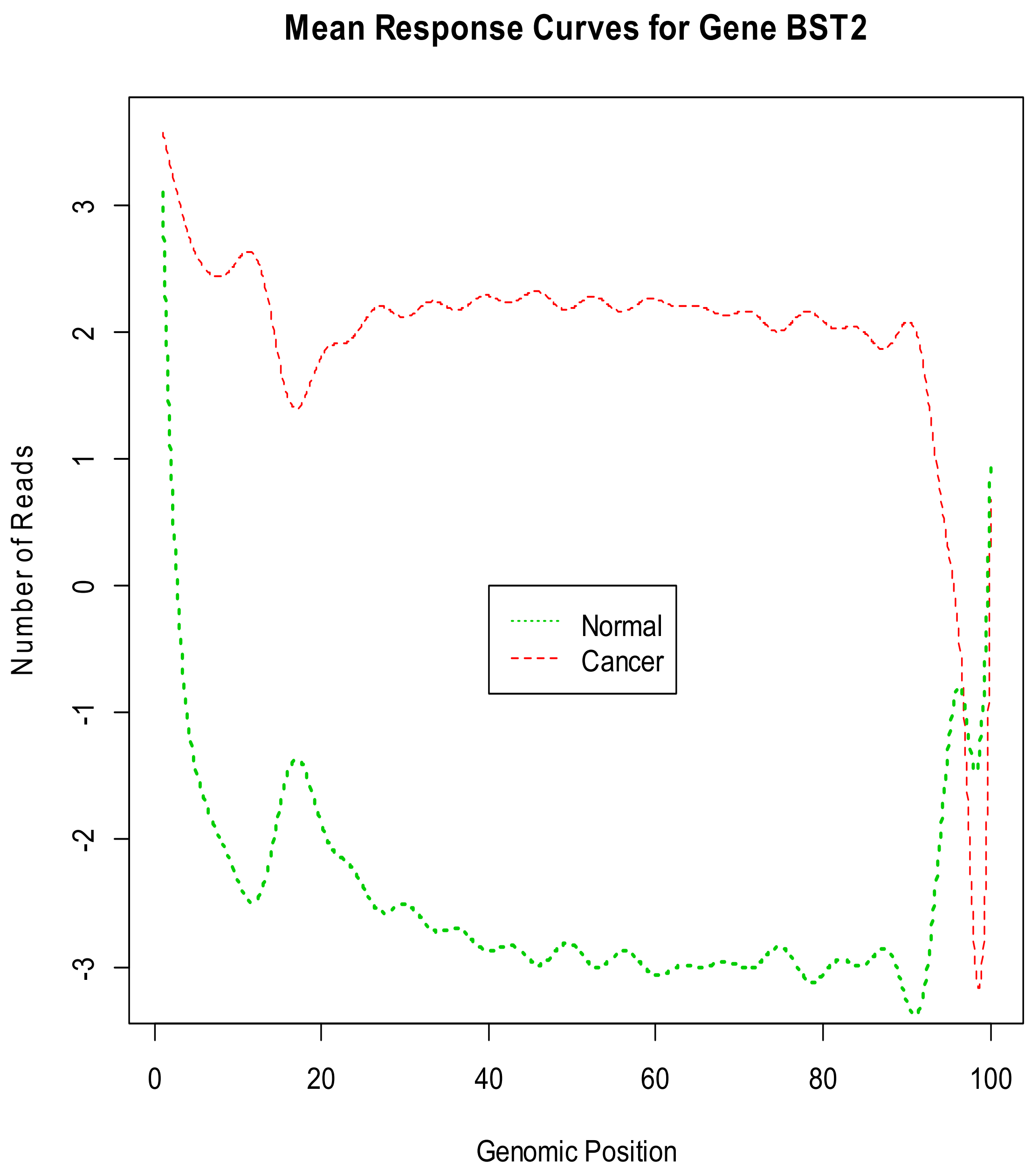

Fig. S2B. Average unit-step response curves of gene BST2 in the tumor and normal samples.

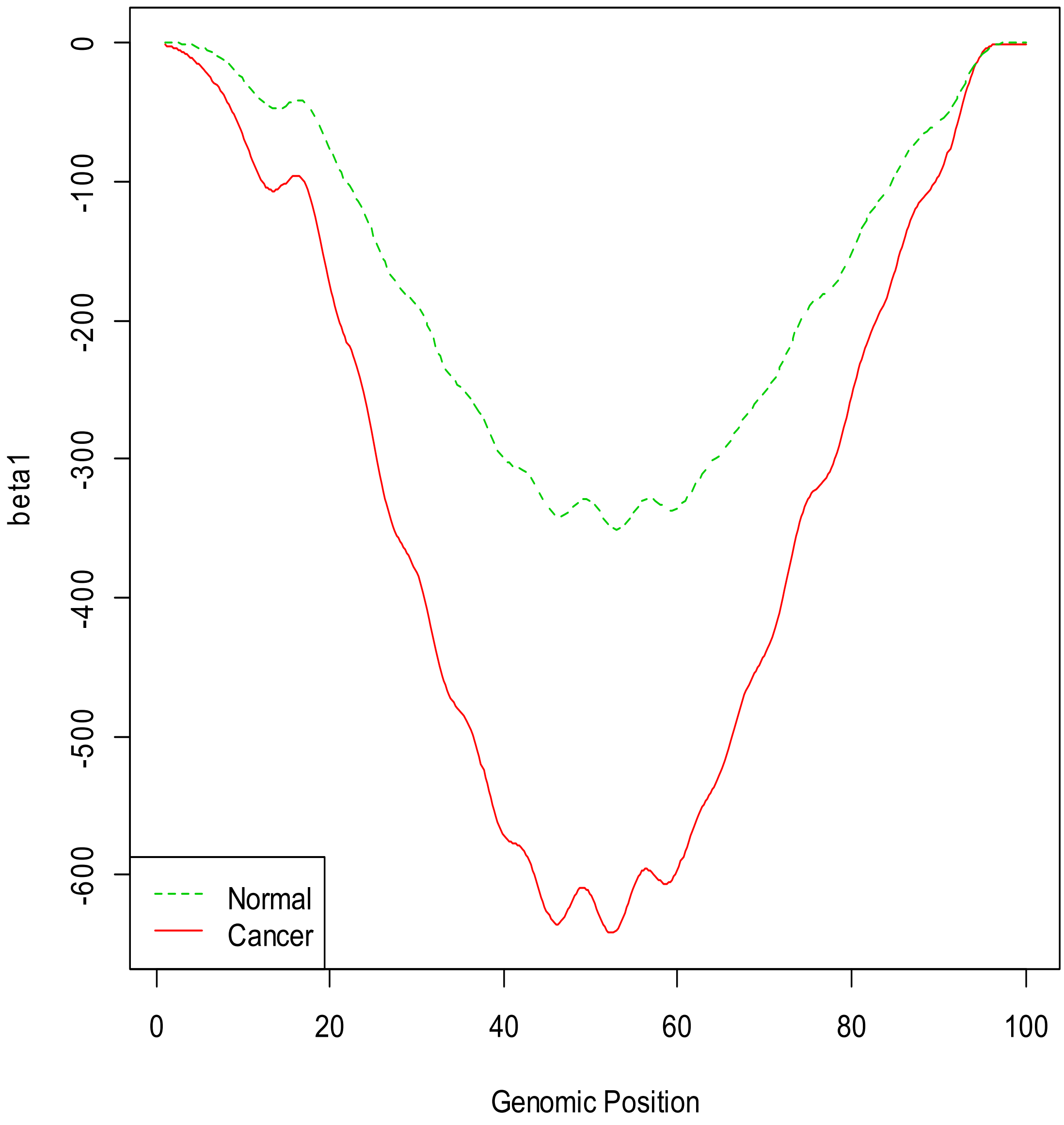

Fig. S2C. Average coefficient curve $\beta_1(t)$ of the ODE for gene BST2.

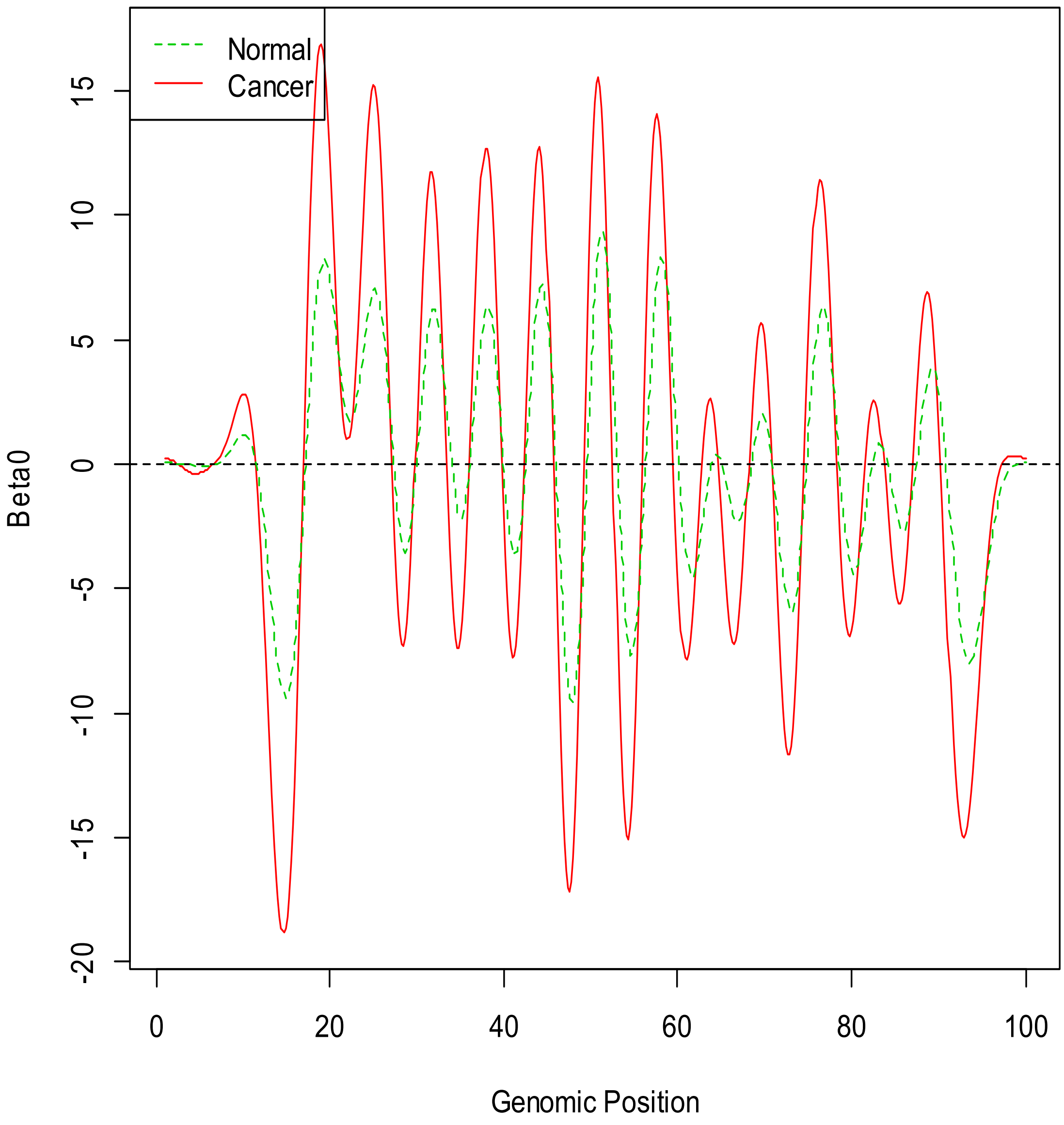

Fig. S2D. Average coefficient curve $\beta_0(t)$ of the ODE for gene BST2.
